# Supplementary material for: Impacts of solar intermittency on future photovoltaic reliability
Source: Nat Commun. 2020 Sep 22;11:4781. doi: 10.1038/s41467-020-18602-6 (PMC7508863; doi:10.1038/s41467-020-18602-6)
Supplement: Supplementary file 1 — Supplementary Information [file 41467_2020_18602_MOESM1_ESM.pdf]

Supplementary Information for

Impacts of Solar Intermittency on Future Photovoltaic Reliability

Jun Yin, Annalisa Molini, Amilcare Porporato

The following figures and tables provide complementary information related to the main text:

Supplementary Figures

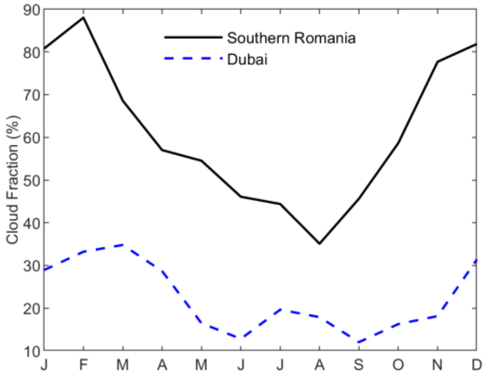

Supplementary Figure 1 Monthly cloud fraction averaged over 2001-2010 from CRU TS v 4.04 data [https://crudata.uea.ac.uk/cru/data/hrg/]. Source data are provided as a Source Data file.

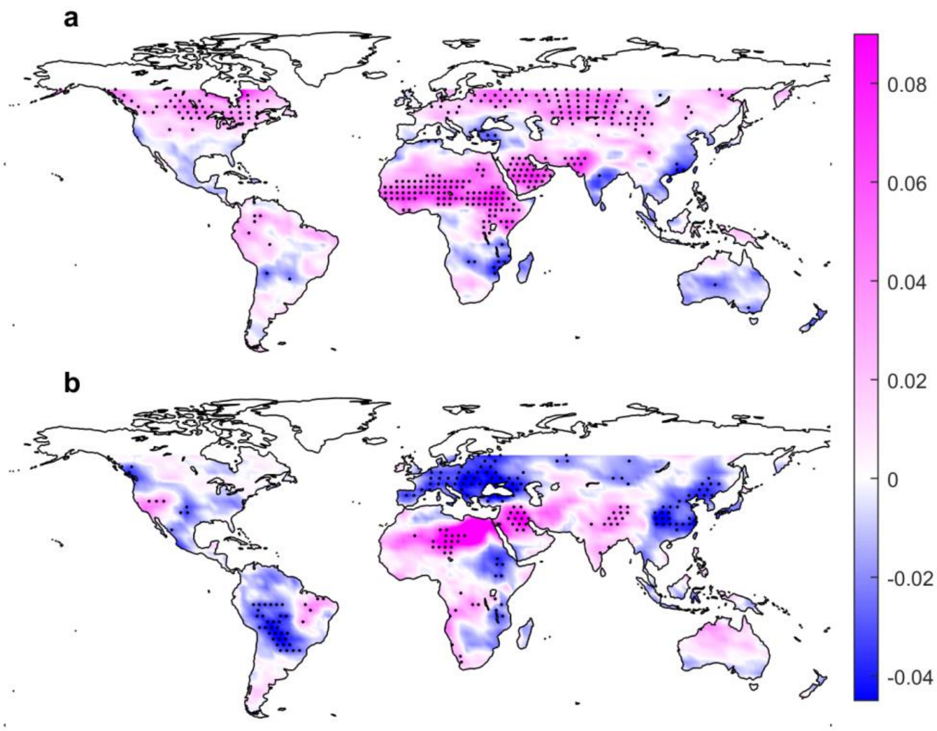

Supplementary Figure 2. Ensemble means of ΔLOLP as in Figure 2 c and d in the main text but for design LOLP of 0.2 in (a) January and (d) July. Source data are provided as a Source Data file.

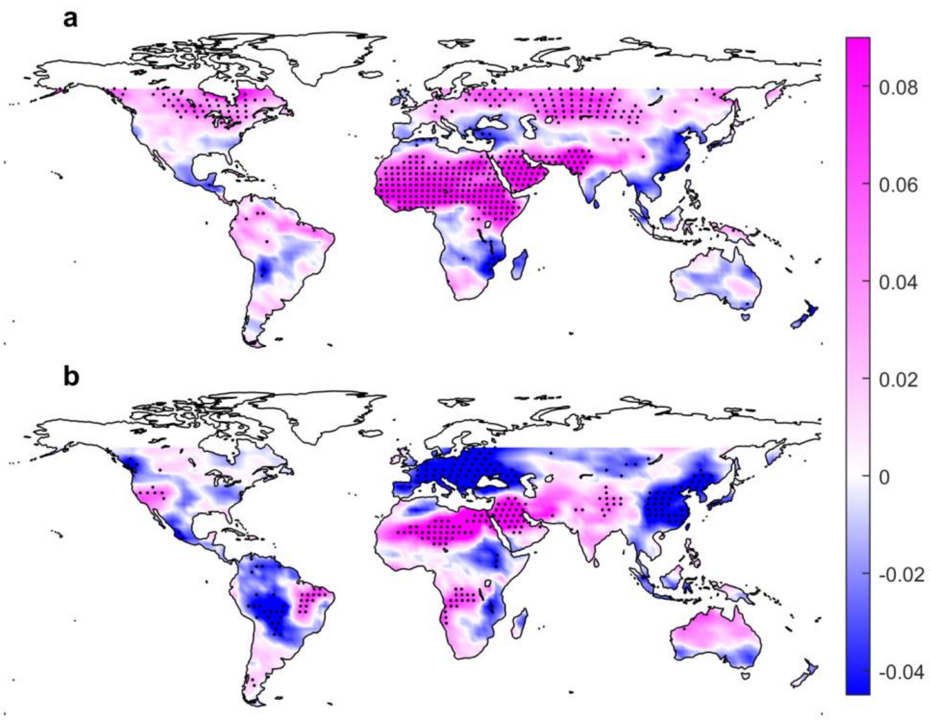

Supplementary Figure 3. Ensemble means of ΔLOLP as in Figure 2 c and d in the main text but for design LOLP of 0.4 in (a) January and (d) July. Source data are provided as a Source Data file.

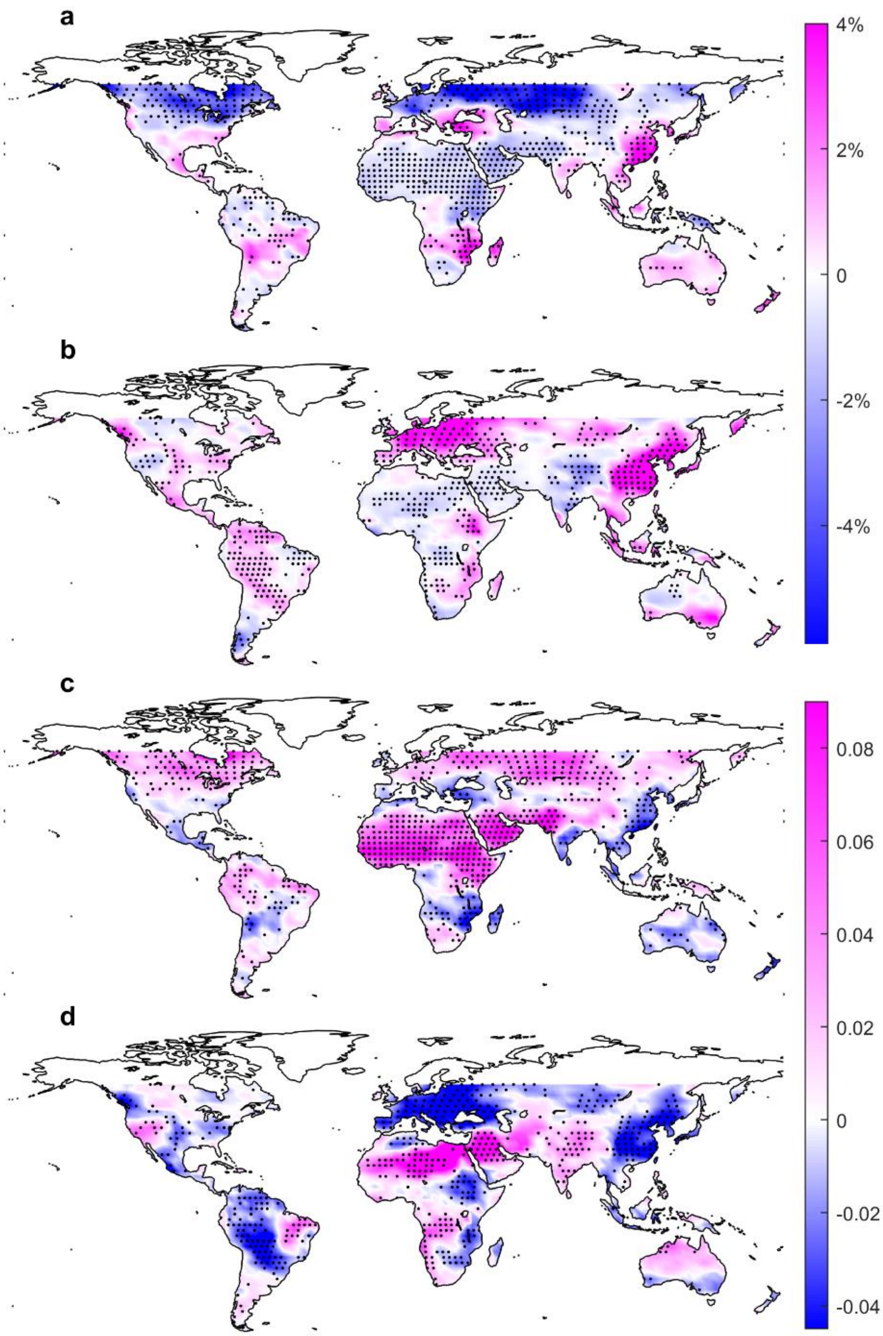

Supplementary Figure 4 As in Figure 2 but the dots show that over eleven climate models eight or more have the same sign of change

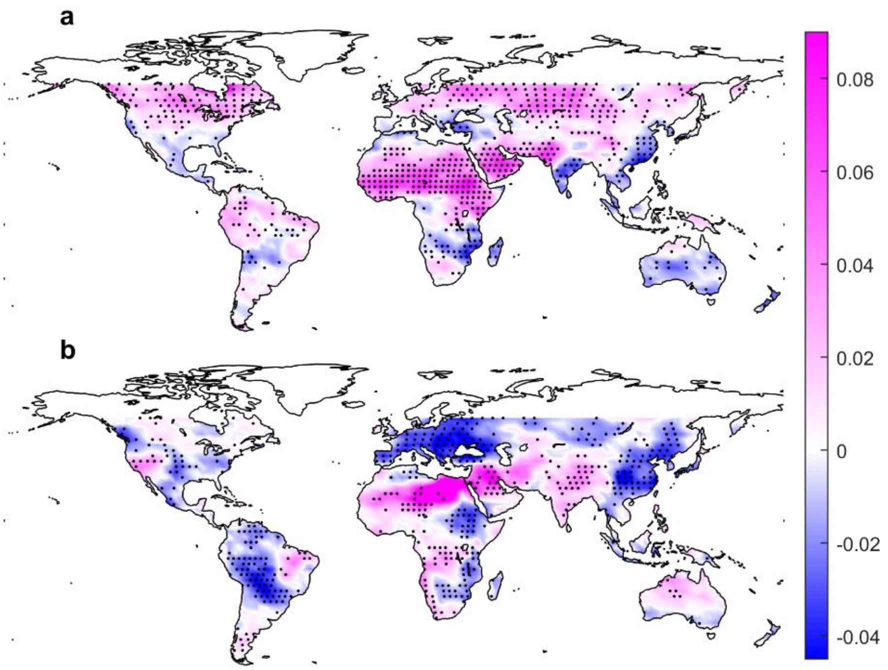

Supplementary Figure 5 As in Supplementary Figure 2 but the dots show that over eleven climate models eight or more have the same sign of change.

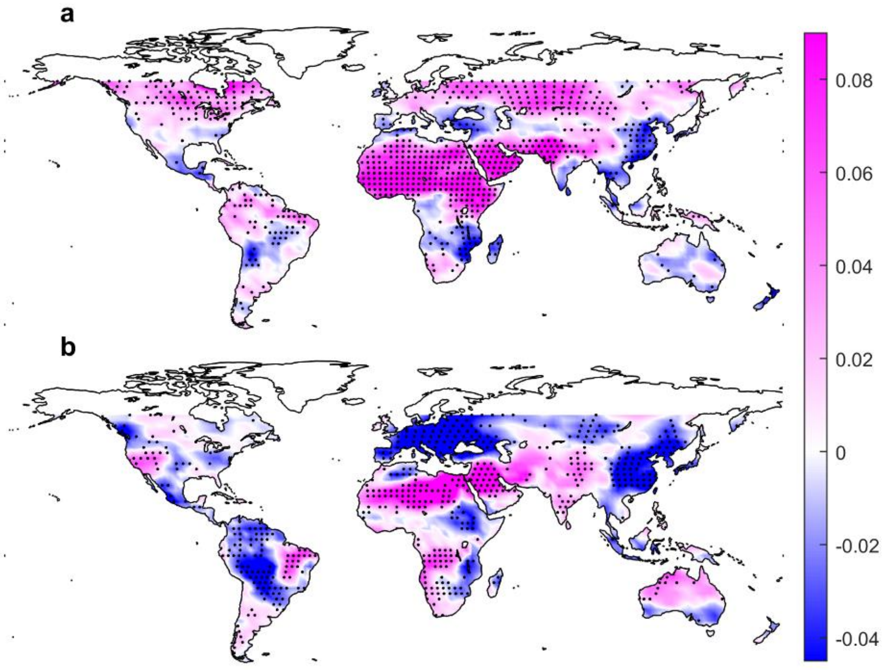

Supplementary Figure 6 As in Supplementary Figure 3 but the dots show that over eleven climate models eight or more have the same sign of change.

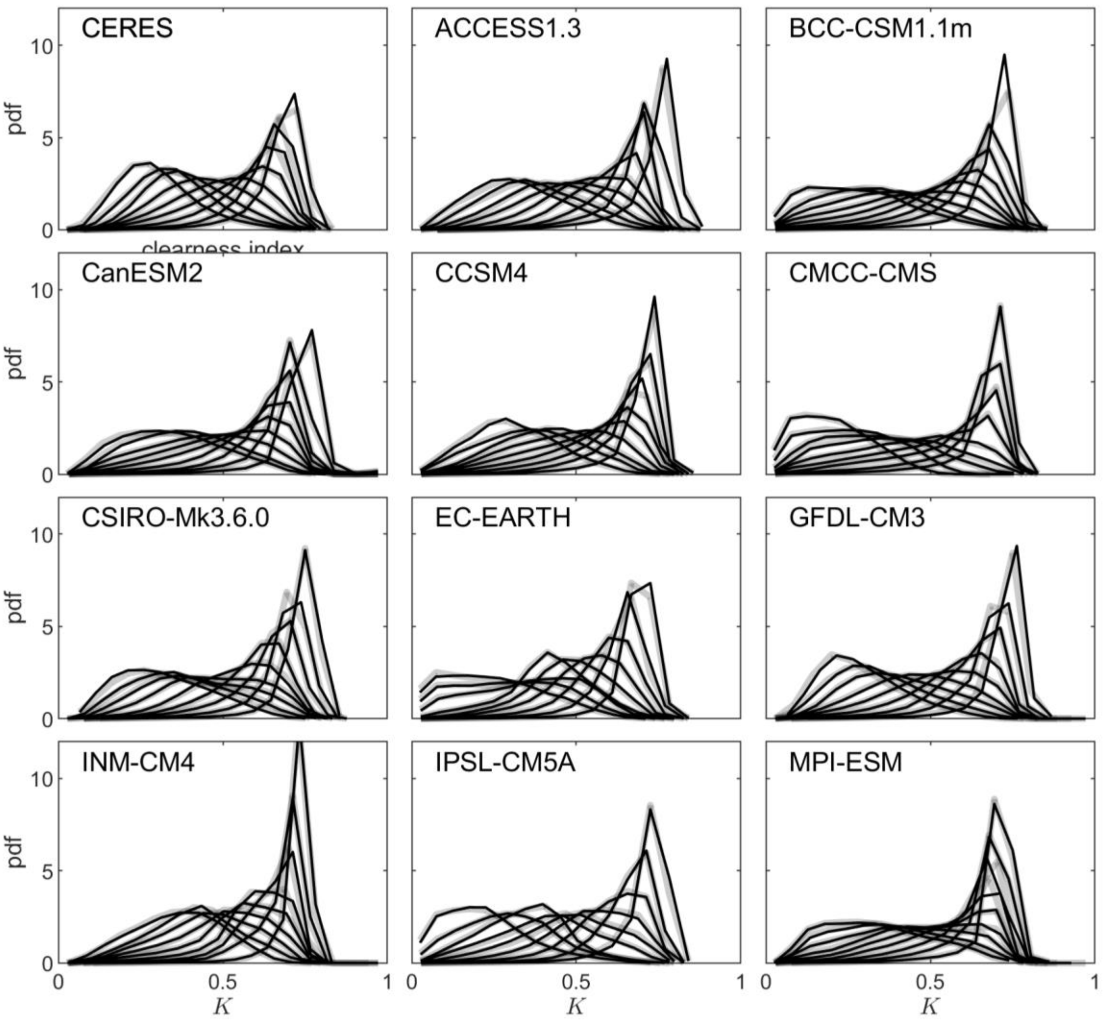

Supplementary Figure 7. The first panel is the same as Figure 3a in the main text and the rest are referred to the distributions of *K* from 11 climate model outputs during 2006-2015 (dark color) and 2041-2050 (light color) in ‘rcp45’ experiment.

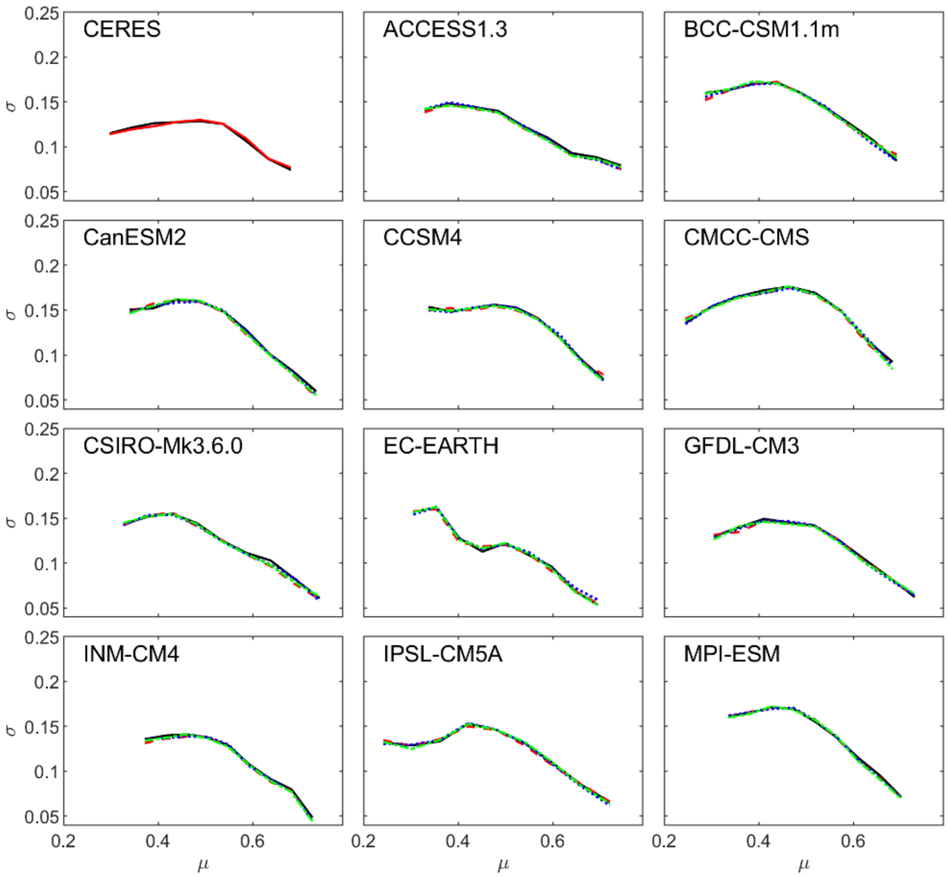

Supplementary Figure 8. $\mu \sim \sigma$ relationship in the month of January. The standard deviation, σ, in regions where the monthly mean is around μ (binning interval of 0.05). In top-left panel, the data are from CERES during 2001-2009 (black line) and 2010-2018 (red line); in other panels, the data are from the corresponding climate model outputs from 'RCP4.5' scenario during 2006-2015 (black line), 2041-2050 (red dash line), 2021-2030 (blue dot line), and 2031-2040 (green dash-dot line).

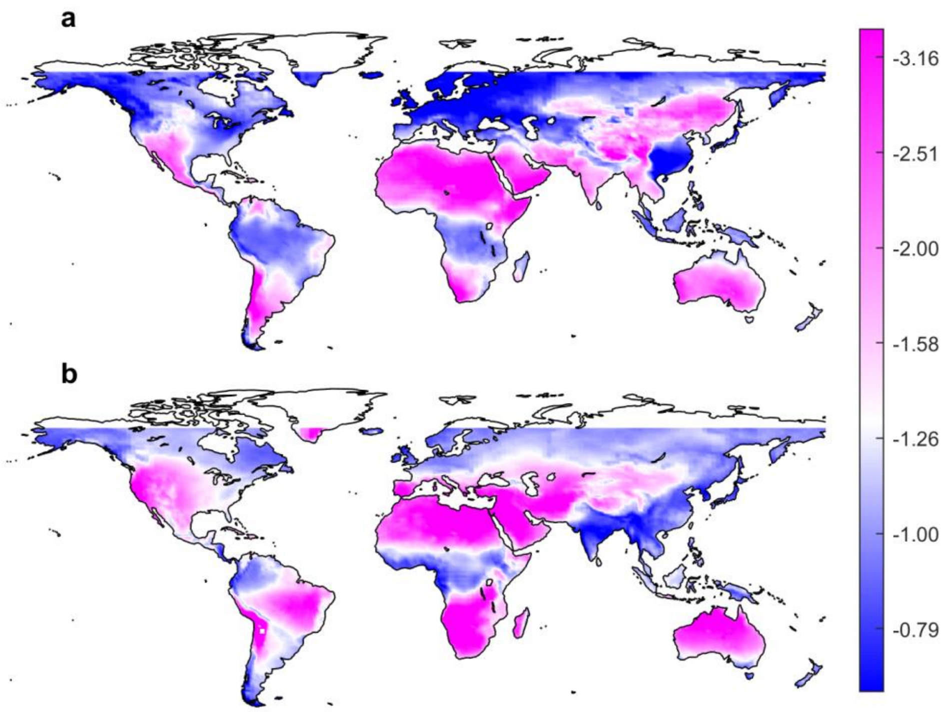

Supplementary Figure 9. As in Figure 5 in the main text but for design LOLP of 0.2.

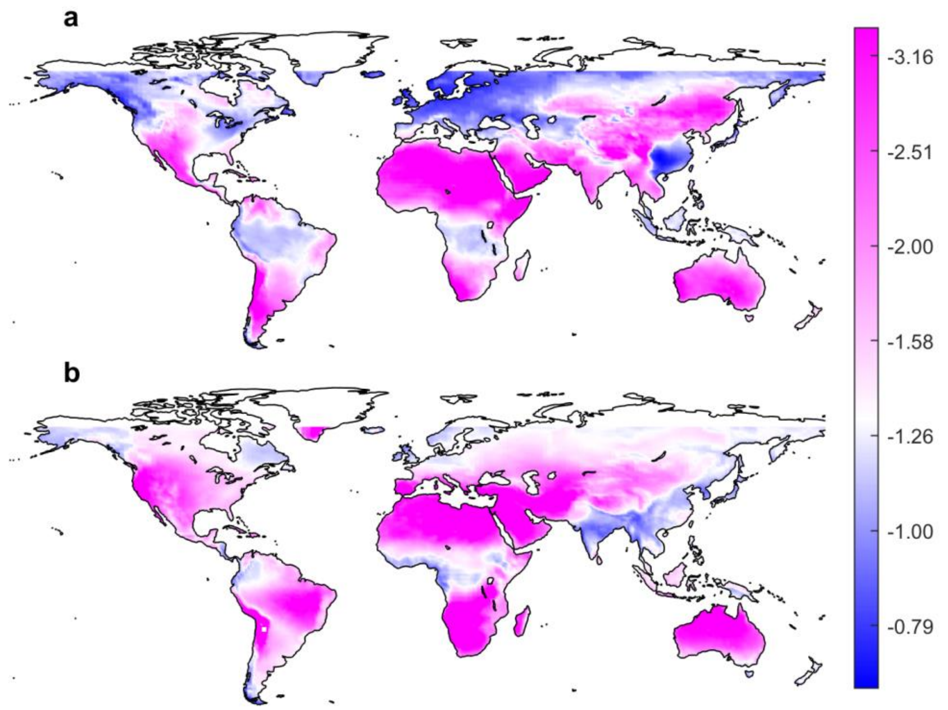

Supplementary Figure 10. As in Figure 5 in the main text but for design LOLP of 0.4.

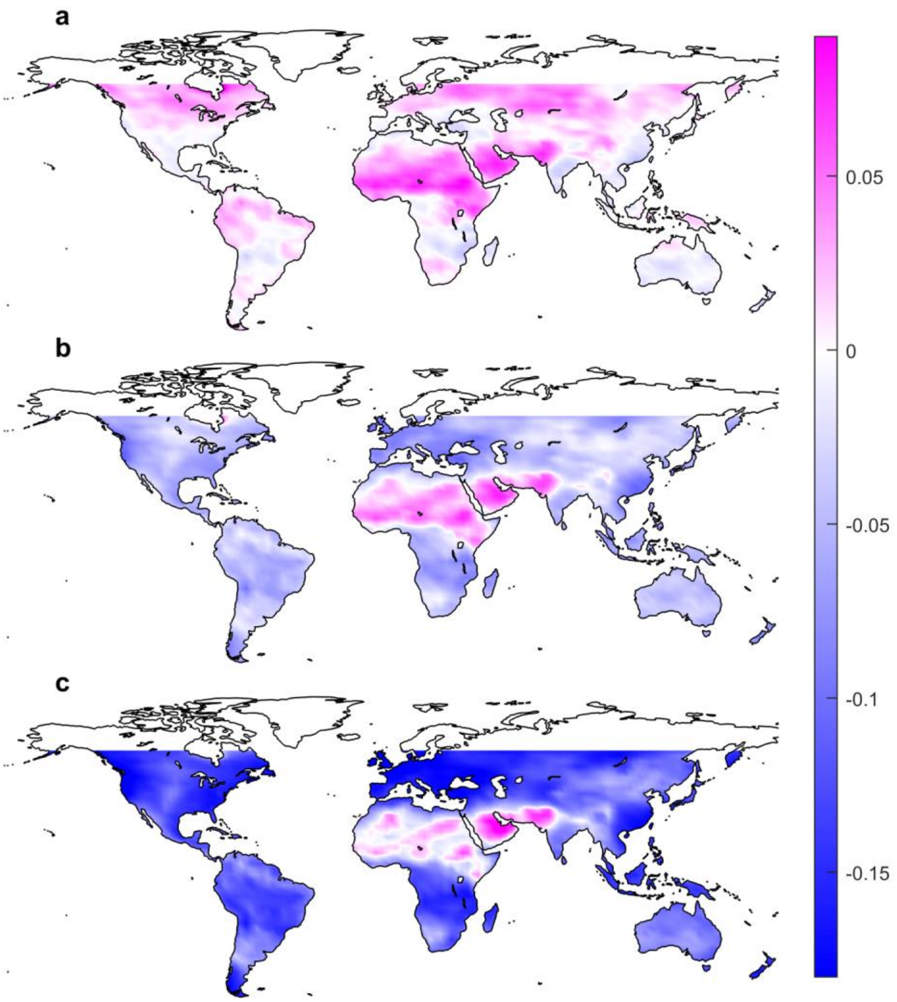

Supplementary Figure 11. Ensemble means of the change of LOLP with (a) no variability mitigation, (b) 25% mitigation, and (c) 50% mitigation from 11 climate model outputs between 2006-2015 and 2041-2050 in the month of January. The LOLP during 2006-2015 (i.e., design LOLP) is set as 0.2.

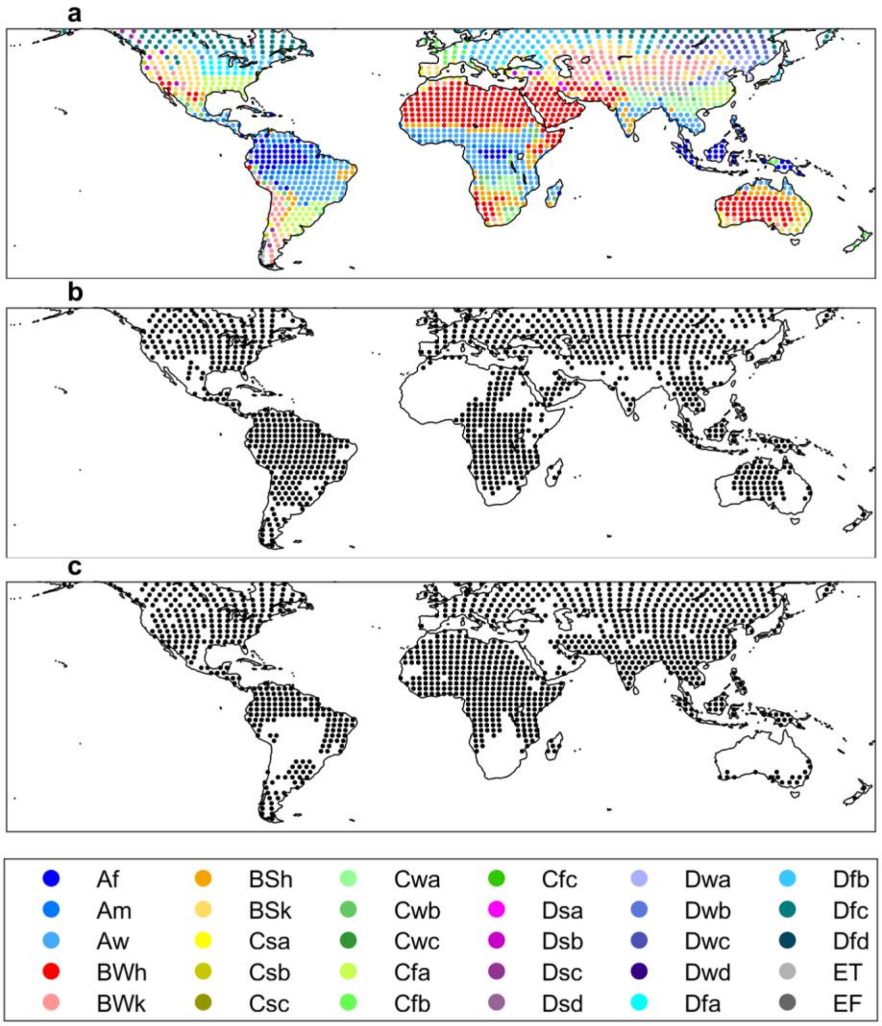

Supplementary Figure 12 (a) Classification of Climate Zones following Beck et al., (2018) (DOI: 10.1038/sdata.2018.214) at 280-km equal-area grids. Different colors represent different climatic zones (see legend and Supplementary Table 2). (b and c) The black dots show that the clearness index comes from beta distributions as confirmed by the Kolmogorov-Smirnov tests at 0.05 significant levels. The clearness index is collected at daily timescale from CERES satellite products (see Methods) during 2010-2018 in the month of January (b) or July (c) at each grid point averaging over an area within 1000 km radius and within the same climatic zone as identified in (a).

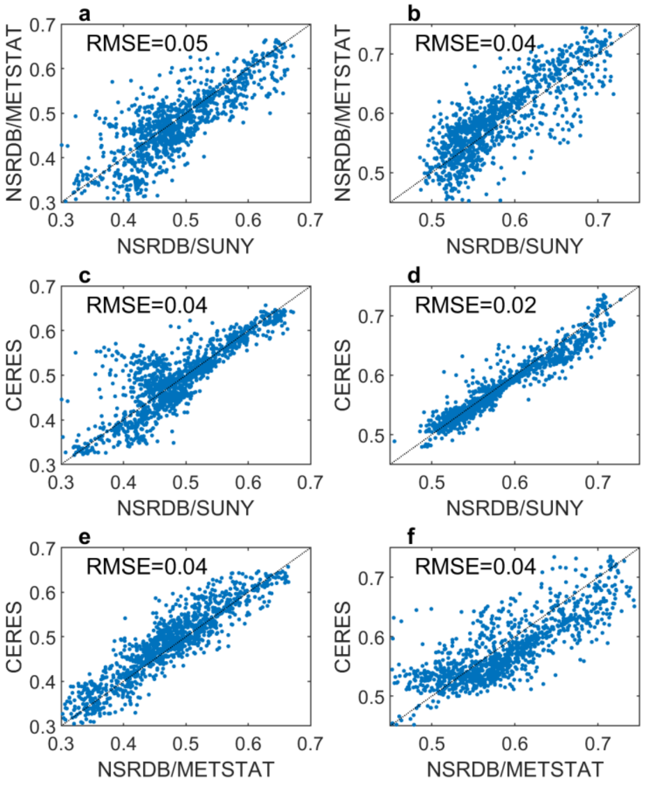

Supplementary Figure 13. Comparison of clear index averaged over 2001-2010 in (a and c) January and (b and d) July between (a and b) SUNY and METSTAT outputs from National Solar Radiation Data Base and between (c and d) SUNY and CERES SYN satellite data. Source data are provided as a Source Data file.

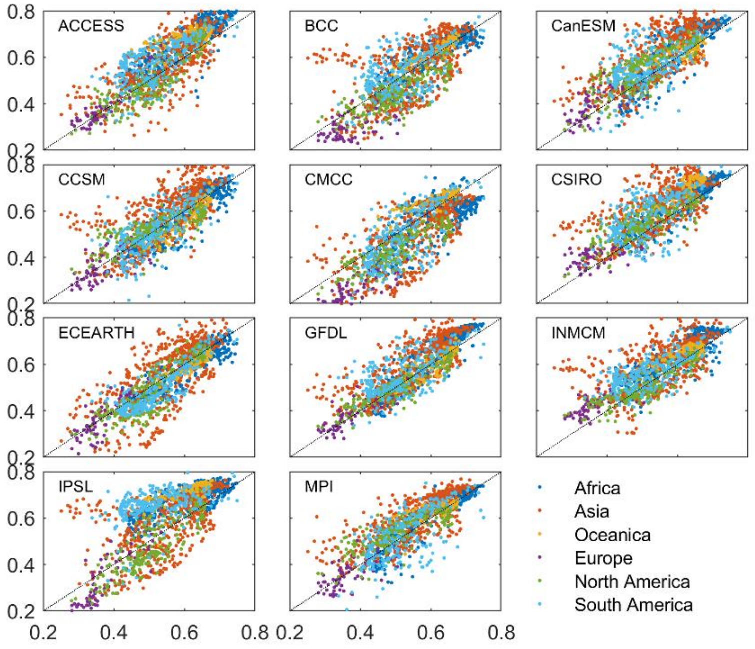

Supplementary Figure 14. Comparison of clear index averaged over 2006-2015 in January between satellite data (x-axis) and climate model outputs (y-axis) from different climate model outputs (model names are shown in the upper right of the panel).

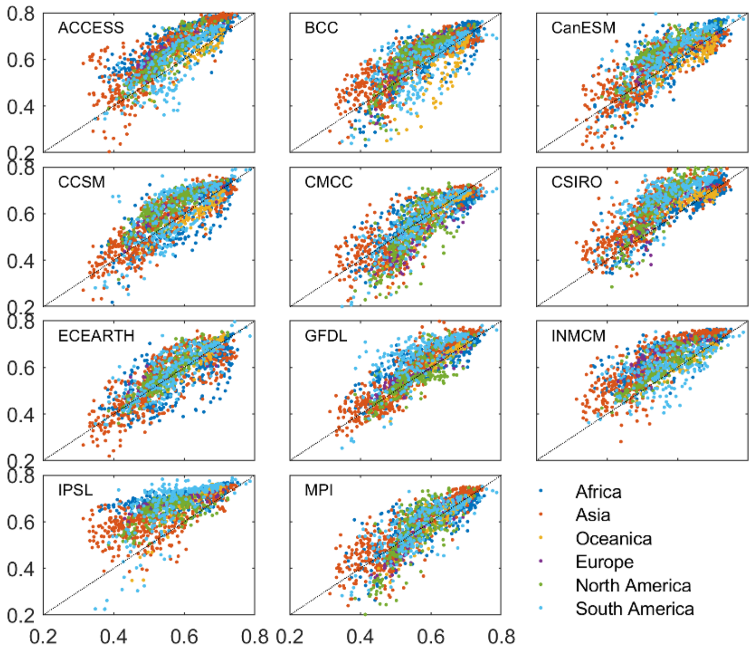

Supplementary Figure 15. As in Supplementary Figure 14 but for the month of July.

Supplementary Tables

Supplementary Table 1. 95% confidence intervals of mean clearness index in the Southern Romania and Dubai, UAE as shown in Figure 1 in the main text.

	Southern Romania		Dubai, UAE	
	January	July	January	July
Current	0.388 ± 0.001	0.549 ± 0.001	0.569 ± 0.001	0.606 ± 0.0003
Future	0.402 ± 0.001	0.580 ± 0.001	0.595 ± 0.001	0.607 ± 0.0003

Supplementary Table 2. Summary of Kolmogorov-Smirnov test results in Supplementary Figure 12.

Climatic Zones	Climate Zone Codes	Total number of grids	Grids passes K-S tests in January		Grids passes K-S tests in July	
			number	(%)	number	(%)
Tropical, rainforest	Af	87	86	98.9	85	97.7
Tropical, monsoon	Am	63	49	77.8	43	68.3
Tropical, savannah	Aw	220	177	80.5	168	76.4
Arid, desert, hot	BWh	283	100	35.3	179	63.3
Arid, desert, cold	BWk	96	84	87.5	66	68.8
Arid, steppe, hot	BSh	104	57	54.8	51	49.0
Arid, steppe, cold	BSk	122	92	75.4	95	77.9
Temperate, dry summer, hot summer	Csa	18	12	66.7	8	44.4
Temperate, dry summer, warm summer	Csb	9	2	22.2	5	55.6
Temperate, dry summer, cold summer	Csc	0	0	-	0	-
Temperate, dry winter, hot summer	Cwa	54	38	70.4	35	64.8
Temperate, dry winter, warm summer	Cwb	20	13	65.0	12	60.0
Temperate, dry winter, cold summer	Cwc	0	0	-	0	-
Temperate, no dry season, hot summer	Cfa	66	35	53.0	58	87.9
Temperate, no dry season, warm summer	Cfb	26	22	84.6	20	76.9
Temperate, no dry season, cold summer	Cfc	0	0	-	0	-
Cold, dry summer, hot summer	Dsa	3	0	0.0	2	66.7
Cold, dry summer, warm summer	Dsb	9	8	88.9	1	11.1
Cold, dry summer, cold summer	Dsc	7	3	42.9	2	28.6
Cold, dry summer, very cold winter	Dsd	0	0	-	0	-
Cold, dry winter, hot summer	Dwa	17	15	88.2	17	100.0
Cold, dry winter, warm summer	Dwb	16	3	18.8	15	93.8
Cold, dry winter, cold summer	Dwc	34	28	82.4	33	97.1
Cold, dry winter, very cold winter	Dwd	0	0	-	0	-
Cold, no dry season, hot summer	Dfa	23	23	100.0	21	91.3
Cold, no dry season, warm summer	Dfb	99	95	96.0	97	98.0
Cold, no dry season, cold summer	Dfc	94	78	83.0	93	98.9
Cold, no dry season, very cold winter	Dfd	0	0	-	0	-
Polar, tundra	ET	40	25	62.5	37	92.5

Polar, frost	EF	0	0	-	0	-
North Temperate Zone (35-66.5N)		494	431	87.3	431	87.3
North Subtropics (23.5-35N)		252	92	36.5	212	84.1
North Tropics (0-23.5N)		322	174	54.0	287	89.1
South Tropics (0-23.5S)		285	260	91.2	160	56.1
South Subtropics (23.5-35S)		130	71	54.6	31	23.9
South Temperate Zone (35-66.5S)		27	17	63.0	22	81.5
All		1510	1045	69.2	1143	75.7

Supplementary Table 3. Root mean square error (RMSE) between climate model outputs and satellite data as shown in Supplementary Figure 14 and Supplementary Figure 15.

	January							July						
	Af.	Asia	Oce.	EU	N.Am.	S.Am.	All	Af.	Asia	Oce.	EU	N.Am.	S.Am.	All
ACCESS	0.07	0.08	0.09	0.04	0.05	0.09	0.07	0.10	0.10	0.03	0.08	0.07	0.07	0.07
BCC	0.04	0.11	0.02	0.10	0.08	0.09	0.07	0.06	0.07	0.10	0.07	0.08	0.08	0.07
CanESM	0.06	0.10	0.04	0.06	0.07	0.10	0.07	0.06	0.07	0.04	0.07	0.08	0.07	0.08
CCSM	0.05	0.09	0.05	0.05	0.06	0.07	0.06	0.06	0.07	0.04	0.07	0.08	0.08	0.06
CMCC	0.07	0.11	0.05	0.13	0.08	0.09	0.08	0.05	0.07	0.04	0.09	0.09	0.06	0.08
CSIRO	0.05	0.11	0.08	0.06	0.06	0.10	0.07	0.05	0.10	0.04	0.06	0.10	0.12	0.08
ECEARTH	0.04	0.10	0.03	0.04	0.06	0.05	0.07	0.08	0.07	0.03	0.04	0.04	0.06	0.07
GFDL	0.05	0.09	0.03	0.04	0.04	0.08	0.06	0.06	0.06	0.02	0.03	0.05	0.10	0.06
INMCM	0.06	0.10	0.05	0.07	0.04	0.08	0.07	0.09	0.09	0.02	0.10	0.06	0.08	0.07
IPSL	0.09	0.11	0.11	0.07	0.07	0.16	0.08	0.13	0.12	0.07	0.14	0.11	0.13	0.09
MPI	0.04	0.08	0.05	0.05	0.06	0.08	0.06	0.06	0.06	0.04	0.05	0.08	0.06	0.07

Supplementary Table 4. Climate Models used in this study and their inclusion of indirect aerosol effects

Acronyms	Model Institutions	Full Indirect Aerosol Effects
ACCESS1.3	Commonwealth Scientific and Industrial Research Organization, Australia	Yes
BCC-CSM1.1(m)	Beijing Climate Center, China	No
CanESM2	Canadian Centre for Climate Modelling and Analysis, Canada	Yes
CCSM4	National Center for Atmospheric Research, USA	No
CMCC-CMS	Euro-Mediterranean Center on Climate Change, Italy	No
CSIRO-Mk3.6.0	Commonwealth Scientific and Industrial Research Organization, Australia	Yes
EC-EARTH	EC-Earth consortium, Europe	No
GFDL-CM3	NOAA Geophysical Fluid Dynamics Laboratory, USA	Yes
INM-CM4	Institute for Numerical Mathematics, Russia	No
IPSL-CM5A	Institute Pierre Simon Laplace, France	No
MPI-ESM-MR	Max Planck Institute for Meteorology (MPI-M), Germany	No
